# DNA Methylation as Clinically Useful Biomarkers—Light at the End of the Tunnel

**DOI:** 10.3390/ph5010094

**Published:** 2012-01-18

**Authors:** Victor V. Levenson, Anatoliy A. Melnikov

**Affiliations:** Department of Radiation Oncology, Rush University Medical Center, 1750 West Harrison Street, Chicago, IL 60612, USA; Email: anatomel@hotmail.com

**Keywords:** DNA methylation, biomarker, cfcDNA, cancer, therapy, MethDet

## Abstract

A recent expansion of our knowledge about epigenetic changes strongly suggests that epigenetic rather than genetic features better reflect disease development, and consequently, can become more conclusive biomarkers for the detection and diagnosis of different diseases. In this paper we will concentrate on the current advances in DNA methylation studies that demonstrate a direct link between abnormal DNA methylation and a disease. This link can be used to develop diagnostic biomarkers that will precisely identify a particular disease. It also appears that disease-specific DNA methylation patterns undergo unique changes in response to treatment with a particular drug, thus raising the possibility of DNA methylation-based biomarkers for the monitoring of treatment efficacy, for prediction of response to treatment, and for the prognosis of outcome. While biomarkers for oncology are the most obvious applications, other fields of medicine are likely to benefit as well. This potential is demonstrated by DNA methylation-based biomarkers for neurological and psychiatric diseases. A special requirement for a biomarker is the possibility of longitudinal testing. In this regard cell-free circulating DNA from blood is especially interesting because it carries methylation markers specific for a particular disease. Although only a few DNA methylation-based biomarkers have attained clinical relevance, the ongoing efforts to decipher disease-specific methylation patterns are likely to produce additional biomarkers for detection, diagnosis, and monitoring of different diseases in the near future.

## 1. Introduction

Within a very short time we have seen the birth and tremendous success of the field of molecular biology; starting with deciphering the structure of DNA and resolving the mystery of the genetic code, which has given rise to genetic engineering, genome-wide sequencing, and has recently culminated in the construction of an artificial life form [1]. While progress of basic biology is obvious, its applications to everyday life and to health care are less impressive as inheritable genetic diseases can nowadays be detected, but remain mostly incurable and early detection of disease is still difficult, even in high-risk populations with well-established genetic abnormalities [2].

Genome malfunctions are probably the root cause of most diseases. Indeed, mutations have been detected in different types of cancer [3,4], so changes in the nucleotide sequence of the genome of cancer cells is well-established. Neurodegenerative diseases are no exception as mutations [5,6,7] and other genetic features (e.g., a specific HLA allele [8]) are linked to multiple sclerosis and facilitate development of this disease. Even infectious diseases are affected by genomic features of the host, for example, the functionality of the toll-like receptor 9, which is responsible for the detection of invading microorganisms, depends on two SNPs [9], while resistance to HIV infection may be determined by a specific haplotype of HLA-G [10].

Functionally important structural changes to the genome have to be reflected in patterns of gene expression, otherwise they will remain silent, and will not change the cell. In this sense any functionally effective change to the genome is by definition reflected in changes in gene expression, so any mechanism of gene regulation can be involved and in addition to genetic changes we have to consider epigenetic alterations, including DNA methylation. This dramatic increase of the proverbial haystack with the unknown disease-causing “needle” has a silver lining, as epigenetic features can be modified in order to reverse disease-defining changes and thus to facilitate treatment [11,12].

In this paper we will address recent developments in DNA methylation studies, particularly those that strive to explore methylation changes causing the disease or induced by it as a means to identify the disease and to estimate its natural course and potential for successful treatment. In contrast to genetic mutations, disease-dependent changes of DNA methylation do not involve changes of the nucleotide sequence, so they may potentially be reversed to restore normal genomic function and expression patterns of a disease-free individual. While this ideal scenario has not been proven yet, treatment-specific changes have been recorded [13]. This observation suggests that either a single drug or their combination may be able to modify DNA methylation, and thus gene expression, to restore the genome to the pattern of a healthy individual.

The vast majority of work on disease-related DNA methylation has been done in cancer [14], but changes in gene expression and thus DNA methylation are a much more general phenomenon observed in different diseases (see the last section). The general principles, however, remain very similar, so most cancer-related examples have much wider applications.

Investigation of methylation patterns depends on the substrate that carries disease-specific methylation features. In this regard analysis of tissue appears to be the most direct approach, although many tissues are difficult to retrieve for testing (e.g., brain, pancreas, lung, *etc.*), so a proxy media is needed. In addition, longitudinal testing to follow treatment-dependent changes in tissues is practically impossible, so for clinical analysis disease-specific genomic DNA has to be collected from other sources. One of them is cell-free circulating DNA in blood plasma (cfcDNA).

## 2. cfcDNA as the Source of Genomic DNA for Analysis

cfcDNA is present in plasma of all humans as 0.5–5.0 kilobase (kb) polynucleotide chains [15]. Its origins are still unclear, but passive release during cell death or active secretion from proliferating cells has been postulated [16,17,18,19]. Newly generated cfcDNA is cleared from blood through the hepato-renal system [20], and it can also be degraded by nucleases in blood. Balance of the production and clearance is sufficient to keep cfcDNA concentration in plasma in the range of a few nanograms per milliliter (ng/mL). Physiologically, cfcDNA is a metabolic derivative of the turnover of cells in the body, so changes in its plasma concentration may reflect presence of pathological processes. It is established that significant cell injury and death raise the levels of cfcDNA. Trauma, cancer, inflammation, stroke, and even extensive physical exercise can induce significant increase in cfcDNA concentration [16,21,22,23,24]. Even more important for its clinical application, cfcDNA contains mutations specific for the primary tumor [25] and DNA methylation patterns characteristic of the disease [23,26]. It appears that diagnosis and monitoring of the disease can be accomplished using cfcDNA from blood plasma.

It has to be noted that cfcDNA from plasma is not the same as cfcDNA isolated from serum. The most obvious difference is higher levels of cfcDNA that can be isolated from serum than from plasma. Apparently, additional DNA in serum is associated with clotting process [27,28], suggesting that cellular DNA from peripheral blood mononuclear cells (PBMC) is released into serum during clot formation [27,28,29,30,31]. Much lower concentrations of cfcDNA in plasma compared to serum probably better reflect cfcDNA in circulation [27,28] and, by extension, better reflect the disease. Release of cellular DNA during clotting may not interfere with analysis of mutations and other genetic markers, where the presence or absence of a marker is sufficient. It will, however, negate any type of quantitative analysis, including analysis of DNA methylation, by masking methylation patterns of cfcDNA and substituting them with patterns of genomic DNA from blood cells.

Cell-free DNA and RNA have been found in cerebrospinal fluid (CSF) [32,33], although detailed analysis and possible clinical applications are still pending. It appears that a relatively invasive nature of CSF collection may become a deterrent for widespread use of this biological fluid, especially for screening purposes.

On the contrary, excretion of cfcDNA through the renal system can be used to collect it in the least invasive manner. Transrenal DNA from pregnant women has been used for analysis of fetal DNA [34,35,36], although reported results are less impressive than the data obtained with cfcDNA from plasma. It appears that technical problems related to high volume and low concentration of cfcDNA in urine, and potential contamination by genomic DNA from epithelial cells of the urinary tract will have to be resolved before the use of transrenal DNA can be expanded beyond detection of specific sequences [20].

## 3. DNA Methylation as a Biomarker

### 3.1. Location

Methylation of promoter regions with high GC content (CpG islands, CGI) is probably the most direct mechanism of regulation, and higher methylation is associated with lower transcriptional activity [37,38,39]. Interestingly, promoter regions are likely to be just one of multiple areas that regulate expression, as methylation of intragenic fragments can also have a significant impact on gene expression [40]. Another, less direct transcriptional regulation takes place through selective methylation of imprinted regions, which define higher-order chromatin structure and thus gene silencing within large genomic regions [41,42]; this type of regulation may play a role in disease development and progression [43]. DNA methylation also plays an indirect role in preserving functional and structural genomic integrity, because some endogenous retroelements contain potentially strong promoters which are heavily methylated and transcriptionally inactive in normal cells. Their de-methylation in cancer cells can lead to promoter activation, abnormal gene expression, and metabolic dysregulation [44,45]. Transcriptionally active retroelements are also capable of genomic translocations potentially leading to disruption of the genome through insertional mutagenesis [46,47].

Different patterns of gene expression are probably the most obvious molecular distinction between normal and cancerous cells. These differences implicitly involve different levels of DNA methylation, and methylation-based biomarkers are actively investigated [48,49,50]. If we define a biomarker as a region with consistent difference between cancerous and normal cells, then the biomarker can be either abnormally hypermethylated or abnormally demethylated in cancer. For example, these differences, which can become biomarkers, can reflect either activation of normally inactive oncogenes, which will correlate with demethylation, or inactivation (and thus hypermethylation) of usually active tumor suppressor genes in cancer cells. This breadth of potential biomarkers has to be recognized as viable possibilities even though the general reduction of global DNA methylation in cancer is well established.

### 3.2. Development of Biomarkers—Approaches and Techniques

The vast majority of attempts to develop diagnostic biomarkers are based on analysis of cancer tissues [51,52,53,54]. Tactically, they can be divided into a candidate-gene approach [54,55] and global analysis [56,57,58,59,60,61,62,63]. A combination of these techniques can also be used; it involves microarray-based genome-wide analysis of gene expression in normal and cancerous cells (with or without treatment with demethylating agents) followed by analysis of methylation in promoters of selected genes that are expressed differently [64,65]. It appears that candidate-gene approach is underpowered and so far has failed to produce accurate biomarkers. The global analysis approach takes advantage of expression profiling to narrow down potential candidates (e.g., [58,59,66]) and has the power of genome-wide testing without preconceived ideas of what gene has abnormal methylation status in cancer cells. It should be noted, however, that a combination of expression microarrays and analysis of individual genes may still have investigator-imposed limits, because in many cases only promoter regions of differentially expressed genes are tested for methylation. As we mentioned above, changes of transcriptional activity could be imposed by methylation of 3' areas or even in intragenic regions, which might be left untested. Apparently, the most unbiased approach for discovery of putative biomarkers among abnormally methylated fragments has to test precisely methylation and do that for the whole genome in a comprehensive manner. At least two approaches can do the job; one of them is microarrays with either a genome-wide library of all CGI [67] or tiling microarrays, while the second is the next generation genome-wide sequencing.

Tiling microarrays (Affymetrix, Roche-NimbleGen, Agilent) are designed to cover the entire genome or selected specific areas and can be one of the best solutions for an unbiased genome-wide testing. They differ by the length of the probes and the average distance between them; in this regard Affymetrix tiling array 2.0 probably allows the most detailed interrogation of the genome, as it is designed with the average distance of 50 bp between neighboring probes and allows testing of the genome at a very high resolution. Since microarrays are used for the detection of differences, methylated and unmethylated sequences have to be either physically separated or to have detectable sequence differences. Methylated fragments can be selectively precipitated with antibodies against methylated cytosine or methylated DNA binding proteins [68,69], or unmethylated fragments can be selectively destroyed by digestion with methylation-sensitive restriction enzymes [56,58,66,70]. Resolution of either approach is insufficient to identify methylation status of every cytosine in the genome, but this precision might be unnecessary for biomarker development, because regulation of gene expression is likely to depend on methylation of genomic fragments rather than an individual cytosine.

Alternatively, genome-wide sequencing can be used to identify abnormal methylation at a nucleotide-based resolution. Currently, it is the only technique that can interrogate every cytosine in the genome. It is important to keep in mind that similar to arrays, sequencing serves to detect methylation-dependent changes in DNA sequence that have to be introduced beforehand. In most cases bisulfite modification [71,72,73] is used to convert unmethylated cytosines to uracils while keeping methylated cytosines intact. This conversion has certain benefits and pitfalls that are discussed in recent reviews [48,74].

### 3.3. Development of Biomarkers—Different Requirements for Different Biomarkers

As tissue-based biomarkers are identified directly in tumor tissue, potential heterogeneity of the analyte is greatly reduced. This does not mean that tissue samples are completely homogeneous. Natural heterogeneity of cancer cells, the presence of different cell types (e.g., stroma) and different sources of abnormally methylated fragments have to be considered [75]. Moreover, field cancerization (or field defect) can produce abnormally methylated fragments in seemingly normal surrounding tissue [76,77,78]. Only a few studies have addressed relationship between clinicopathological characteristics of the tumor (e.g., cancer type, stage, grade, and size) and field defect in the surrounding tissue [79,80,81], so using it as a healthy counterpart in all cases to compare methylation patterns with the tumor may be risky. In addition, comparison of healthy tissue and tumors has no value for development or clinical use of screening biomarkers, and may have only limited value for diagnostic biomarkers, because tissue sampling is usually very difficult or even outright impossible. At the same time, tissue-based biomarkers can be very useful for prognosis or for prediction of response to treatment [82,83,84,85].

Prognostic biomarkers should reflect the risk of recurrence [83], while predictive biomarkers the probability of response to a specific therapy [86]. It is clear from this difference that predictive biomarkers are tightly linked to a specific drug or drug combination or even the whole treatment regimen, including chemo- and radiotherapy. It remains to be seen whether predictive biomarkers developed for monotherapies will be useful for multifactorial therapeutic regimens. On the other hand, prognostic biomarkers should reveal the outcome of the disease, thus they should reflect tumor properties (e.g., its aggressiveness) that are somewhat less dependent on the course of therapy. Thus, tissue-based prognostic and predictive biomarkers provide a time-frozen snapshot of the disease that can be used to select therapy and assess probable outcomes. On the other hand, they can be useless if recurrent disease develops new characteristics, including changes in drug sensitivity or emergence of new mutations.

With the exception of circulating tumor cells (CTCs), blood-based biomarkers are only indirectly linked to the disease, but can be assessed again and again over time. This feature is essential to monitor disease changes, including its sensitivity to therapy, and to detect emerging recurrence as early as possible. Blood is one of the most accessible biological fluids that has close contact with all tissues and can carry disease-specific substances. This makes blood the media of convenience, where different biomarkers; for detection, diagnosis, prediction of response, and prognosis of outcomes can be identified. From a practical perspective blood analysis of different markers is a well-established clinical routine [87,88], so once the appropriate biomarker is developed, the infrastructure for blood testing is already in place and only minimal changes will be required to integrate the biomarker into clinical practice.

### 3.4. Blood-Based DNA Methylation Biomarkers for Detection and Diagnosis

The major problem in development of DNA methylation-based biomarkers from blood is rather basic. The concentration of cell-free circulating DNA is frequently very low (5–10 ng/mL), so usually only a few genes can be interrogated. To circumvent this problem methylation of cellular DNA (from PBMC) in multiple genes is tested (e.g., [89,90,91]) or, if cfcDNA is used, only a few investigator-selected genes are analyzed [92,93,94,95]. Unfortunately, the link between abnormal methylation in PBMC and in primary tumors has not been unequivocally established, so much work is needed before PBMC-based methylation assays will enter the mainstream. Selection of particular genes for analysis in cfcDNA, on the other hand, is fraught with potential for investigator-induced bias reducing the probability to develop a precise biomarker.

**Figure 1:**
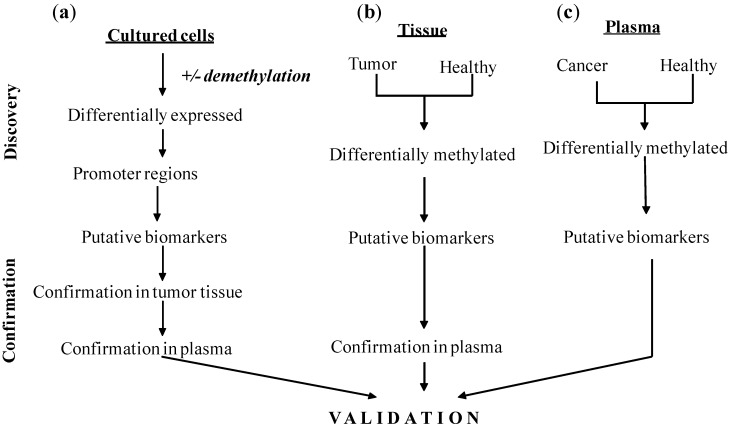
Different approaches for discovery of biomarkers based on genome-wide assessment. (**a**) Genome-wide expression patterns in cultured cells are compared, and a set of differentially expressed genes is identified. Promoters of these genes are then tested for abnormal methylation, and a group of putative biomarkers is selected for tissue analysis. Promoters confirmed in tissues are then investigated in cfcDNA. (**b**) Genome-wide methylation patterns in cancer tissue are compared to patterns in healthy tissue, and differentially methylated fragments are selected as putative biomarkers. These fragments are tested in cfcDNA. (**c**) Genome-wide patterns of methylation are compared using cfcDNA from cancer patients and healthy controls. Differentially methylated fragments are selected as putative biomarkers.

Three alternative approaches allow significant expansion of the targets that can be tested as biomarkers (Figure 1). One of them is the previously mentioned pharmacologic unmasking of the promoter region by treatment with demethylation agents and analysis of re-expression of previously suppressed genes using genome-wide microarray analysis [96,97]. While this approach allows unbiased identification of re-expressed genes, the procedure requires testing of cells in culture rather than analysis of actual tumors. In addition, selection of a specific regulatory sequence within a gene for assessment of methylation is still subjective. Moreover, this technique cannot identify genes that are abnormally demethylated in cancer cells as their expression will not perceptibly change after treatment with demethylation agents. In practice, this technique requires three steps. First, an appropriate cell line has to be used to apply demethylating agents, then potential candidates have to be selected and confirmed first in tumor tissues, and then in cfcDNA. Effectively, three different objects are analyzed by this approach with the hope that candidates selected in cultured cells will be confirmed in cfcDNA.

Another approach has been used by investigators at Epigenomics, Inc to develop a cfcDNA-based biomarker for colorectal cancer. They left out expression changes in cell culture, and started directly with well-characterized tumor tissues. Using a combination of genome-wide scanning [98] with methylated CpG island amplification [99] and differential methylation hybridization [56], they identified three fragments in colorectal tumors that were further tested in cfcDNA from plasma of patients with this type of cancer [100]. One of the fragments, located in the promoter of SEPT9, is currently being tested for FDA approval. The latest results of PRESEPT clinical trial indicate that the SEPT9 assay has 67% sensitivity and 88% specificity [101] for detection of colorectal cancer, which is much better than other established techniques (e.g., fecal occult blood test, FOBT), but still leaves one third (33%) of cancer patients undetected while 12% of healthy individuals are mis-diagnosed. Abnormal expression and/or methylation of SEPT9 has been observed in breast [102,103], ovarian [104,105], head and neck [106,107], and other human tumors [108], indicating that a one-gene biomarker might be sub-optimal.

If the transition of tissue-identified putative biomarkers to plasma determines imperfect performance of SEPT9, the entire approach “from tissue to plasma” can be flawed regardless of the number of genes in the biomarker. Several studies suggest that this may indeed be the case; our analysis of DNA from tumor and cfcDNA from plasma of patients with ovarian adenocarcinoma has shown that methylation patterns in cfcDNA are similar, but not identical to those in tumor tissues [109]. These results have been independently confirmed by others in studies with hepatocellular carcinoma [110,111], suggesting that tumor tissue-derived biomarkers are unlikely to perform equally well in plasma.

What can be done to analyze genome-wide methylation and select biomarkers by direct analysis of cfcDNA despite its low abundance in blood? One recently developed technique combines bisulfite modification and whole genome amplification (quantitative Methylation Analysis of Minute DNA amounts after whole Bisulfitome Amplification, qMAMBA) [112]. While its performance for genome-wide investigation remains to be tested, results from analysis of five selected fragments suggest that this technique can be used for whole genome analysis. Another technique (methylation detection, MethDet) depends on digestion of unmethylated fragments by a methylation-sensitive restriction enzyme; it has been developed for the genome-wide analysis in our laboratory [113]. The goal of MethDet analysis is to identify cfcDNA-based diagnostic biomarkers that can be used to detect and distinguish different diseases. Indeed, different cancer-specific patterns of methylation in cfcDNA have been found using the proof-of-principle platform designed to test methylation in 56 genes in each sample [23,26,109,114,115]. In addition, we have shown that the removal of a primary tumor causes a specific change in the cancer-specific pattern, while another change reflects therapy [13]. Importantly, even with the proof-of-principle MethDet platform we have identified disease-specific patterns in patients with inflammatory and benign diseases [23,26], and with pre-cancerous conditions [116]. It appears that specific changes in methylation of cfcDNA can indeed differentiate various types of solid tumors and detect them before they become invasive.

Following this logic on a genome-wide scale, we have developed a biomarker panel to detect patients with pancreatic adenocarcinoma and differentiate them from patients with chronic pancreatitis. While the clinical version of the test is still in validation, the biomarker has already demonstrated its significant potential.

### 3.5. Blood-Based DNA Methylation Biomarkers for Other Diseases

Psychiatric and neurological*—*While a lot of work targets oncology-related methylation-based biomarkers in cfcDNA, it can also be used in other areas of medicine. An elegant paper presented recently by the Patsalis group describes excellent results of a clinical trial for methylation-based detection of trisomy 21 in blood of pregnant women [117]. In brain tissue of patients with psychiatric diseases, including schizophrenia and bipolar disorder, specific methylation patterns have been detected as well [118,119,120], suggesting that analysis of methylation in cfcDNA can produce blood-based biomarkers for these diseases. Our work with cfcDNA isolated from patients with multiple sclerosis (MS) indicates that patterns of MS are different from those of healthy controls even when the disease is inactive, and the MS patients are in remission [115]. These results suggest that a genome-wide search can produce a precise blood-based biomarker for the detection of MS. Significantly, methylation pattern in cfcDNA of MS patients changes during the attack and is quite dissimilar from the pattern observed in remission, so a blood-based biomarker for detection of attacks is likely to be feasible as well [115]. Considering that 90% of attacks in MS are asymptomatic, and thus no anti-inflammatory treatment is administered to these patients, detection of asymptomatic attacks through regular testing of methylation in cfcDNA can have a significant impact on MS management.

Cardiovascular—abnormal methylation in blood has been detected in individuals at high risk for this disease [121,122,123,124], although it is still unclear whether these abnormalities are linked to high level of cholesterol in blood, or if they are independent. Cross-sectional studies in individuals with different types of cardiovascular disease and different levels of cholesterol may be needed before clinical importance of observed differences can be established.

Infectious disease is another example of methylation changes associated with physiological change, in this case after viral [125,126,127] or bacterial [128] infection that activates some cellular genes while suppressing others. Additional work is needed to explore whether methylation patterns are pathogen-specific or are a general sign of infection. In either case changes of patterns over the time of infection can give essential insights into the time course of change and maybe even shed some light on the sources of cfcDNA.

Psychological stress is also reflected in changes of DNA methylation patterns [129,130], suggesting that DNA methylation biomarkers can identify many physiological or pathophysiological changes in humans.

### 3.6. Blood-Based DNA Methylation Biomarkers for Monitoring of Drug Treatment

If DNA methylation is a reflection of human physiology, it can be expected to reflect environmental factors, including diet, as well as physiological changes induced by drugs. Indeed, there is evidence that environmental factors, e.g., exposure to lead [131] or particulates in air [132], influence epigenetic features, including DNA methylation (reviewed in [133]). This evidence suggests that substances, specifically designed to affect selected areas of human physiology (*i.e*., drugs) will change DNA methylation patterns in a particular, drug-specific manner.

There is a number of methylation-specific drugs that have been developed to directly affect DNA methylation [134,135,136], so demethylation of the genome and activation of gene expression are unsurprising. More interesting are the selective non-nucleoside demethylation agents—hydralazine and procainamide [137,138] that have the potential to selectively effect methylation in certain genes. These possibilities await genome-wide assessment of methylation providing additional avenue of research.

Taking advantage of the treatment modalities for multiple sclerosis (disease-modifying drugs are usually used as a monotherapy) we have evaluated DNA methylation patterns in cfcDNA from MS patients undergoing treatment with Avonex^®^, Tysabri^®^, and Copaxone^®^. Results of this study, done with the small proof-of-principle platform for assessment of 56 promoters in each sample, indicate that every drug induces its own methylation pattern, which is different from either the patterns of healthy controls or of untreated MS patients. When these observations are confirmed in a longitudinal study, the results will open the possibility of cfcDNA methylation-based monitoring of treatment and, ultimately, for prediction of response to a specific drug or a therapeutic regimen.

## 4. Conclusions

It appears that many if not all changes in human physiology may be reflected in a specific pattern of cfcDNA methylation. Amazingly, the origin of cfcDNA itself is still a matter of intense investigation with several potential mechanisms being considered. Specific methylation patterns may be produced by a limited subset of cells undergoing cell death in response to a particular stimulus, be it a change in environment, or development of a disease. Alternatively, this DNA may be generated during normal cellular lifecycle (“metabolic DNA” [15,17]) or as a part of active horizontal gene transfer [139,140,141]. If that is indeed the case, dissemination of disease via genometastasis might be possible [142] with truly frightening implications. Furthermore, at any given time cfcDNA may be produced by a combination of different mechanisms, and their importance may vary depending on particular conditions. It is also quite possible that the assumed homogeneity of cfcDNA may be a figment of our ignorance. Additional investigations are obviously required to resolve the mysteries of cfcDNA. We hope that these studies will facilitate a more targeted development of various biomarkers for clinical use.
